# The prevalence of chronic kidney disease–associated pruritus and its impact on quality of life in hemodialysis patients: a commentary from experts from two countries

**DOI:** 10.1093/ckj/sfae003

**Published:** 2024-01-04

**Authors:** Emilio Sanchez-Alvarez, Marian Goicoechea, Antoine Lanot

**Affiliations:** Hospital Universitario de Cabueñes, Department of Nephrology, Gijón, Spain; Hospital General Universitario Gregorio Marañón, Nephrology Unit, Madrid, Spain; Normandie Université, Unicaen, CHU de Caen Normandie, Néphrologie dialyse et transplantation, Côte de Nacre, Caen, France

To the Editor,

In patients undergoing hemodialysis, chronic kidney disease–associated pruritus (CKD-aP) is a condition that has a high impact on quality of life (QoL). Using data from cohorts from past decades, some authors establish a prevalence of CKD-aP of 40%–90% in patients undergoing hemodialysis and 19%–29% in non-dialyzed patients [1], and clinicians still tend to underestimate its burden [2, 3]. More current data suggest a prevalence of around 40% in moderate or severe forms of pruritus [4].

In recent years, interest in CKD-aP has increased; thus, in European countries such as Spain and France, different initiatives have been launched to understand its prevalence and how it affects quality of life, and diagnostic and treatment algorithms have been proposed. In Spain, a cross-sectional study [5] investigated the severity, prevalence and impact on QoL of CKD-aP. A short *ad hoc* survey was designed, including items of validated tools for pruritus assessment, such as Question 20 of the Kidney Disease Quality of Life questionnaire, Worst Itch Numeric Rating Scale (WI-NRS) and the Itch Severity Scale. Originally designed to test QoL in dialysis patients, this survey includes a question (Question 20) that evaluates itching in the past 4 weeks. This question has been the basis of the largest international studies of CKD-aP prevalence in dialysis to document the prevalence of pruritus over the past 20 years [3]. The survey was distributed to all Spanish Society of Nephrology members visiting patients with advanced CKD. Nephrologists visiting patients with advanced CKD asked them to voluntarily answer the questionnaire. A total of 1605 patients voluntarily answered the questionnaire. According to the WI-NRS questionary, the prevalence of CKD-aP was 50.5%, and 26.7% of patients presented moderate to severe CKD-aP [5].

At the same time, a prospective multicenter observational study analyzed the prevalence of CKD-aP in a cohort of 1304 French hemodialysis patients who were systematically screened for pruritus (Pruripreva study) [6]. The prevalence of moderate to very severe pruritus (defined as a WI-NRS score ≥4) was 23.5%. Before the study initiation, pruritus was unknown by physicians in 37.6% of these affected patients, and 56.4% of them were treated. Itch impact on QoL was analyzed according to severity (WI-NRS) through the 5-D itch scale, the EQ-5D and SF-12 questionnaires. Regardless of the QoL domains tested with these three validated tools, the more severe the pruritus, the more significant the impact on QoL [6].

With the intention of determining the concern about and perception of CKD-aP among Spanish nephrologists, a previous survey was conducted through an anonymous survey [7]. Of the 135 participating nephrologists, 114 (84%) considered underdiagnosis a fact. In their centers and regarding the rate of diagnosed CKD-aP, 57 (42%) indicated that it was 0%–10%, 56 (42%) 0%–20%, and only 22 (16%) reported that its prevalence is >20%. Forty percent believed that diagnosis occurs because the patient refers to it, and only 27% because the physician asks for it [7]. Similarly, the French Society of Nephrology conducted an online survey to describe the management of CKD-aP by its members, and 100 questionnaires were analyzed. The three main messages of this survey were (i) French nephrologists underestimated the prevalence and relevance of CKD-aP, (ii) the severity of CKD-aP was not systematically assessed despite its significant impact on QoL and (iii) the effectiveness of current therapeutic options was not satisfactory [8].

Recently, some algorithms have been published for the screening, diagnosis, assessment and treatment of CKD-aP among patients undergoing hemodialysis [9, 10]. Both algorithms include 24-h WI-NRS and Self-Assessed Disease Severity questionnaires. In Spain, Buades *et al*.’s algorithm was designed by a multidisciplinary panel that included dermatologists [10].

Difelikefalin, a selective, peripherally restricted kappa opioid receptor agonist recently approved for the treatment of moderate to severe pruritus in patients undergoing hemodialysis [11], is recommended as the first treatment option for patients in dialysis in both algorithms as it is the only approved product for CKD-aP in Europe.

The identified discrepancy between the reality and perception (Table 1) of the prevalence of CKD-aP indicates a continued underdiagnosis, probably due to a lack of proactive approaches as a result of unclear guidance for its management and ineffective or off-label therapeutical options with limited evidence. These new algorithms will help us have a more proactive attitude, identifying those patients who suffer from CKD-aP via a systematic screening of pruritus; in addition, they provide us with relevant information about whom to treat and how, and bring QoL to CKD patients in need.

**Table 1:** Evidence of the CKD-aP prevalence reported by patients and nephrologists.

			CKD-aP prevalence	
Author/year	Countries	Sample size (*N*)	Patient reported	Nephrologist reported
Aresté *et al*. 2023 [5]	Spain	1605	50.5% total, 26.7% moderate to severe	
Lanot *et al*. 2023 [6], Pruripreva study	France	1304	23.5% moderate to very severe	
Rayner *et al*. 2017 [12], DOPPS registry	20 countries^a^	6256 patients, 268 nephrologists	19.5% moderate, 18% very much or extremely bothered	65% estimated that <5% of their patients suffered from severe pruritus; nephrologists underestimated the prevalence of pruritus in 69% of facilities
Goicoechea *et al*. 2023 [7]	Spain	135		Only 16% clinicians stated prevalence ≥20%
Touzot *et al*. 2022 [8]	France	100		Median 10.0% (IQR 6.3–17.2)

^a^Australia, New Zealand, Belgium, Canada, China, Bahrain, Qatar, Kuwait, Oman, Saudi Arabia, United Arab Emirates, Germany, Italy, Japan, Russia, Spain, Sweden, Turkey, the UK and the USA.

IQR: interquartile range.
